# Changes in Choroidal Thickness and Structure in Preeclampsia with Serous Retinal Detachment

**DOI:** 10.3390/jcm12020609

**Published:** 2023-01-12

**Authors:** Ayumi Fukui, Hiroshi Tanaka, Nobuhiro Terao, Kenji Nagata, Akifumi Matsumoto, Natsuki Kusada, Kentaro Kojima, Chie Sotozono

**Affiliations:** Department of Ophthalmology, Kyoto Prefectural University of Medicine, 465 Kajii-cho, Hirokoji-agaru, Kawaramachi-dori, Kamigyo-ku, Kyoto 602-0841, Japan

**Keywords:** choroidal thickness, choroidal vascularity index, hypertensive chorioretinopathy, serous retinal detachment, preeclampsia, pregnancy

## Abstract

Preeclampsia is a pregnancy-specific syndrome characterized by hypertension and proteinuria. We retrospectively investigated the clinical features, including choroidal layer thickness and luminal area to stromal area ratio, in a case series of preeclampsia with serous retinal detachment (SRD). The subjects were pregnant women with SRD during hospitalization for preeclampsia from October 2014 to June 2021. Based on medical records, affected eyes, time of onset, fundus examination findings, and subfoveal choroidal thickness (SCT), the choroidal layer thickness and choroidal vascular index (CVI) in each patient was examined. Thirteen eyes from seven patients (mean age 30.7 ± 4.7 years) were included in the study. In all cases, SRD improved without topical ocular treatment. The mean SCT at the initial visit was 424.4 ± 70.5 μm, and all patients had choroidal thickening, which significantly decreased to 286.0 ± 57.9 μm (*p* < 0.01) at the last visit. The mean choroidal inner layer was 162.7 ± 69.4 μm at the initial visit and 122.3 ± 35.5 μm at the final follow-up visit (*p* = 0.06), showing no significant difference; however, the mean choroidal outer layer was 261.7 ± 47.6 μm at the initial visit and 163.7 ± 37.1 μm at the final follow-up visit (*p* < 0.01), thus showing a significant decrease. The mean CVI was 67.2 ± 1.3% at the initial visit, yet it had significantly decreased to 65.4 ± 1.1% (*p* < 0.01) at the final follow-up visit. The findings of this study show that SRD with preeclampsia is associated with increased thickening of the choroidal outer layer, especially in the choroidal luminal area.

## 1. Introduction

Preeclampsia reportedly occurs in approximately 5% of pregnant women [1]. It is a pregnancy-specific syndrome characterized by hypertension and proteinuria [2] that leads to hypertensive retinopathy [3,4] and hypertensive choroidopathy [4,5]. Typical fundus findings in cases of hypertensive choroidopathy, also known as hypertensive chorioretinopathy, include serous retinal detachment (SRD), Elschnig’s spots, and Siegrist streaks. SRD is characterized by subretinal fluid leaking from the choroid onto the retinal pigment epithelium (RPE) due to choroidal circulatory failure. Elschnig’s spots are perimacular yellow spots that are considered to be necrosis of the RPE. Siegrist streaks are linear pigmented stripes on the choroidal artery. Previously, hypertensive choroidopathy was thought to be caused by impaired choroidal perfusion [6]. Recently, it has been reported that choroidal hyperperfusion is involved in hypertensive choroidopathy, as choroidal blood flow velocity correlates with choroidal thickness in patients with hypertensive choroidopathy [7,8]; however, it should be noted that a consensus has yet to be reached. It has also been reported that preeclampsia can sometimes cause SRD [9,10,11], although the clinical features and pathogenesis in such cases remain unclear.

Recent remarkable technological innovations in ophthalmic examination equipment have dramatically elucidated the pathogenesis of retinal [12,13] and choroidal [14,15] diseases. Although the choroid, which is deeper than the retina, cannot be observed via spectral-domain optical coherence tomography (SD-OCT), both enhanced depth imaging OCT (EDI-OCT) and swept source OCT (SS-OCT) can be used for evaluation of the choroid [16] and sclera [17,18] in detail. Hence, the pathogenesis of various choroidal diseases has now become clear. Furthermore, each layer of the choroid as well as the area percentage of vessels and stroma can be evaluated, as the choroid is mainly composed of the large choroidal vessel layer (Haller’s layer), the medium-sized choroidal vessel layer (Sattler’s layer), and the choriocapillaris. Recent studies have proposed the concept of pachychoroid-related disease, which is characterized by thickening of the Haller’s layer and thinning of the Sattler’s layer and choriocapillaris [16]. On the other hand, it has been reported that the Haller’s layer thins with aging [19].

The choroidal vascular index (CVI) is a relatively new parameter for quantitative evaluation of choroidal vascularity [20], and is calculated from OCT-obtained images. It is defined as the ratio of the area of blood vessels to the total area of the choroid, and is considered an important indicator for determining whether the choroidal thickening is luminal or stromal dominant [21].

The purpose of this study was to investigate the clinical features, including choroidal layer thickness and luminal area to stromal area ratio, in a case series of preeclampsia with SRD.

## 2. Materials and Methods

### 2.1. Patients

This retrospective case series study involved pregnant women with hypertensive choroidopathy with SRD who were admitted at the Department of Obstetrics, Kyoto Prefectural University of Medicine (KPUM), Kyoto, Japan for preeclampsia between October 2014 and June 2021, and who were subsequently referred to the Department of Ophthalmology at KPUM due to vision loss. Hypertensive choroidopathy was diagnosed based on the presence of an acute or chronic hypertensive fundus and SRD of the macula, which improved with blood pressure reduction. Based on the medical records of these cases, we examined patient age, the affected eye, the time of onset, complications, the refractive error, and fundus findings. Systolic blood pressure (SBP), diastolic blood pressure (DBP), best-corrected visual acuity (VA) (BCVA), subfoveal choroidal thickness (SCT), choroidal layer thickness, and CVI were compared between those at initial presentation and at the final follow-up visit.

### 2.2. Measurements

BCVA was measured using the Japanese standard Landolt VA chart, and then converted to the logarithm of the minimum angle of resolution (logMAR). In 5 cases, SCT was measured using SS-OCT (DRI OCT Triton^™^; Topcon Corporation, Tokyo, Japan), and it was defined as the distance between the outer border of the hyperreflective RPE and the inner scleral border beneath the center of the fovea. Measurement of the choroidal layer thickness was performed using the method suggested by Branchini et al. [22]. A large choroidal vessel was defined as a lumen of at least 100 μm in diameter within the choroid, and the largest choroidal vessel lumen within 750 μm of either side of the fovea was used for the measurement. A line horizontal to this lumen was drawn at the innermost margin of the large vessel lumen, and a line perpendicular to that horizontal line extending to the inner-most border of the scleral was drawn, which gave Haller’s layer as the choroidal outer layer. Another perpendicular line extending to the outer border of the hyperreflective RPE was drawn, which gave Sattler’s layer and choriocapillaris as the choroidal inner layer, and those boundaries were determined by two retinal experts (HT and AF). In two cases (Case No. 4 and Case No. 6), SCT could not be measured because SD-OCT (3D OCT-2000; Topcon Corporation) was used.

**Figure 1:**
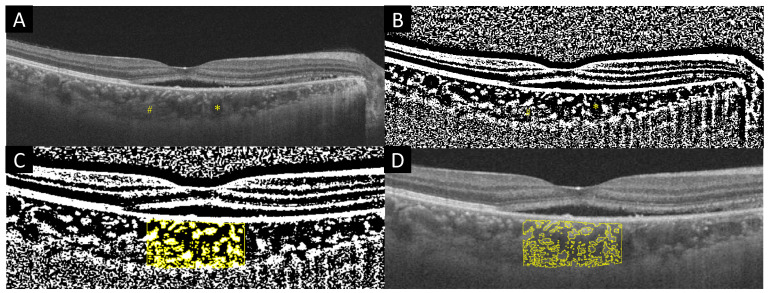
(**A**) OCT image of a preeclampsia patient. The luminal areas (asterisk) and stromal areas (sharp) can be seen. (**B**) Binary image using Niblack method. The luminal areas are darker and the stromal areas are brighter than in (**A**). (**C**) The yellow region is the stromal areas. (**D**) Overlay of the original EDI-OCT image and binarization image.

CVI can be obtained by binarizing choroidal images. The procedure for binarization of the choroidal images is described below. Image binarization was performed using the modified Niblack method with ImageJ software (version 1.47; provided in the public domain by the National Institutes of Health, Bethesda, MD, USA; http://imagej.nih.gov/ij/ (accessed on 7 February 2022)), based on previous reports [20,21]. The subfoveal choroidal area with a width of 1.5 mm at the center of the macula constituted the region of interest. Briefly, OCT images were converted to 8-bit, and Niblack automatic local thresholding was applied to binarize the images. This allows for a more accurate selection of the subfoveal choroid area. With the upper border marked at the RPE and the lower border at the line of light pixels at the choroid scleral junction, the choroidal area was selected using a polygon tool. The images were then converted again to RGB images, and the luminal area was determined using the threshold tool. The dark pixel areas were separated as choroidal luminal areas and the bright pixel areas as stromal areas (Figure 1). CVI was measured by dividing the choroidal luminal area by the total choroidal area. CVI was measured only in cases in which the two experts noted above determined that the subcentral choroidal image was clearly identifiable.

### 2.3. Statistical Analysis

All statistics were performed using RStudio software (RStudio Team (2022). RStudio: Integrated Development Environment for R, PBC, Boston, MA, USA). Statistical significance was assessed with the Wilcoxon-signed-rank test. A *p*-value of <0.05 was considered statistically significant.

## 3. Results

### 3.1. Patient Characteristics

This study involved thirteen eyes (six bilateral and one unilateral [left eye]) of seven patients. The mean patient age was 30.7 ± 4.7 years (range 24–37 years). In all patients, the baby was delivered by scheduled cesarean section, and SRD was observed before cesarean section in two cases and after cesarean section in five cases, and all five patients were aware of vision loss and visual field defects within 2 days after cesarean section. The mean number of weeks of gestation with SRD was 33 weeks and 6 days (range 27 weeks and 5 days to 38 weeks and 6 days). Some patients had complications such as multiple pregnancies, HELLP syndrome, and systemic lupus erythematosus (SLE). The patient with SLE (Case No. 1) was taking prednisolone 5 mg/day at the onset of SRD. In all cases, treatment of hypertension had already begun at the time of the initial ophthalmology examination. The last follow-up visit was an average of 4.6 ± 3.6 months after the initial visit. At the last visit, all patients were seen post-delivery, and blood pressure had lowered without treatment for hypertension. SBP and DBP, respectively, decreased from 144.3 ± 10.3 and 89.9 ± 14.9 mmHg at the initial visit to 123.4 ± 3.4 and 77.7 ± 7.4 mmHg at the last visit (*p* = 0.022, *p* = 0.036). Mean BCVA was −0.06 ± 0.16 (logMAR) at the initial visit and −0.12 ± 0.05 (logMAR) at the last visit, thus indicating no significant difference (*p* = 0.283). In regard to the spherical equivalent refractive error, all patients had normal to moderate myopia and none had hyperopia (Table 1). Of the thirteen eyes, fundus examination findings revealed Elschnig’s spots in six eyes (46.1%), Siegrist streaks in three eyes (23.1%), intraretinal hemorrhage in seven eyes (53.8%), intraretinal edema in four eyes (30.8%), and SRD with a septum in five eyes (38.5%) (Table 2). In all cases, SRD disappeared quickly without ophthalmologic intervention and with recovery of the general condition.

### 3.2. SCT, Choroidal Layer Thickness and CVI

**Figure 2:**
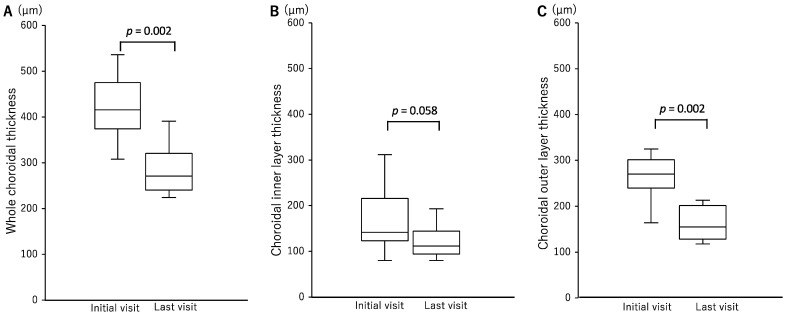
Graphs showing the comparative change in choroidal layer thickness between the initial visit and the last visit. Whole choroidal thickness (**A**) and choroidal outer layer thickness (**C**) significantly decreased at the last visit compared to those at the initial visit. There was no significant difference in choroidal inner layer thickness between the initial visit and the last visit (**B**).

**Figure 3:**
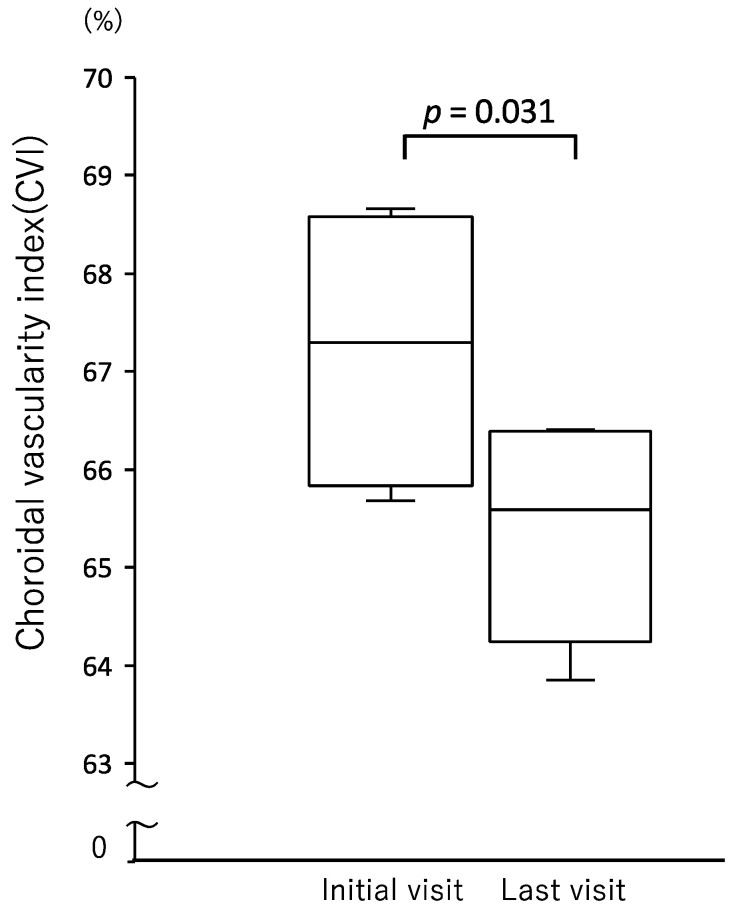
CVI, the ratio of luminal area to choroidal area, was compared between the initial and last visit. CVI was significantly lower at the last visit than at the initial visit.

**Figure 4:**
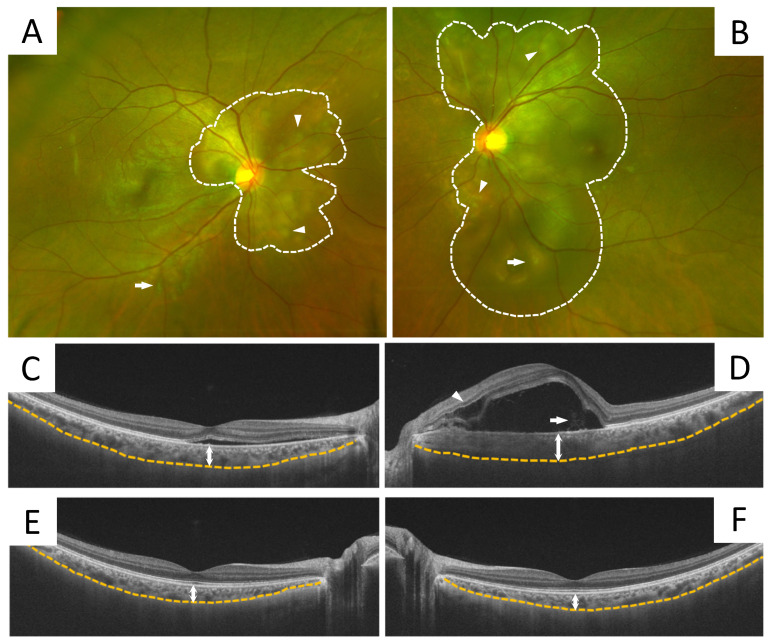
Images showing the findings in a typical case, i.e., a 31-year-old female patient who underwent a scheduled cesarean section at 33 weeks and 2 days and presented at the Department of Ophthalmology complaining of bilateral blurred vision at 1 day post-operative. She had complications of preeclampsia and HELLP syndrome, and was hospitalized in the Department of Obstetrics. Findings of the fundus examination performed at the initial visit revealed SRD (dotted line), Elschnig’s spots (arrowheads), and Siegrist streaks (arrows) around the optic nerve papillae in both eyes (**A**,**B**). OCT examination at the initial visit showed SRD with intraretinal edema (arrowhead) and septum (arrow), and increased SCT in both eyes (OD: 400 μm, OS: 455 μm) (**C**,**D**). After about 3 weeks, her general condition stabilized and her blood pressure normalized. Then, without ophthalmologic intervention, the SRD disappeared and the SCT decreased (OD: 256 μm, OS: 240 μm) (**E**,**F**).

We were able to measure SCT in 10 eyes from five patients (Table 2). The mean SCT was 424.4 ± 70.5 μm at the initial visit and 286.0 ± 57.9 μm at the last follow-up visit (*p* = 0.002) (Figure 2). The mean choroidal inner layer was 162.7 ± 69.4 μm at the initial visit and 122.3 ± 35.5 μm at the last visit (*p* = 0.058), while the mean choroidal outer layer was 261.7 ± 47.6 μm at the initial visit and 163.7 ± 37.1 μm at the last visit; this shows that the thickness of the outer layer at the last visit was significantly less than that at the initial visit (*p* = 0.002) (Figure 2). CVI was measurable in six eyes from three patients. At the initial visit, mean CVI was 67.2 ± 1.3%, yet it had significantly decreased to 65.4 ± 1.1% at the last visit (*p* = 0.031) (Figure 3). The clinical course of a representative case is shown in Figure 4.

## 4. Discussion

In this retrospective case series study involving the evaluation of choroidal layer thickness at the onset and disappearance of SRD in pregnant women with preeclampsia, our findings revealed that there was thickening of the choroid at onset, especially in the choroidal outer layer, and that this thickening also improved when SRD improved. Previous reports have shown that SCT is significantly thicker in pregnant women in the last trimester when compared with that in age-matched healthy non-pregnant women [23]. Although it has been reported that there is no significant difference in SCT during pregnancy [24,25], a consensus has yet to be reached. It has also been reported that pregnant women with preeclampsia in the last trimester have a thicker SCT than healthy pregnant women and non-pregnant women [26,27]. In this study, the SCT was thickened at the onset of SRD, and as the pregnant women’s general condition recovered, the SRD disappeared and the SCT decreased without topical ocular treatment. Kim, et al. [26] and Sharudin, et al. [27] reported a mean SCT of 389.79 μm and 370.7 μm, respectively, in pregnant women with preeclampsia; however, in this present study it was 424.4 μm. It should be noted that this present study involved pregnant women with preeclampsia, and that all the patients had hypertensive choroidopathy with SRD, which may have increased the SCT compared with that reported in the previous studies.

In addition, the CVI findings in this study revealed that the choroid was thickened significantly on the luminal areas. Hypertensive retinopathy is well known, yet hypertensive choroidopathy, which is associated with acute increases in blood pressure, has been reported less frequently. Hypertensive choroidopathy reportedly can occur in cases of preeclampsia [24,28,29,30], renal failure [30,31,32], adrenal cancer [33], and malignant hypertension [34], etc. Until recently, it was considered that hypertensive choroidopathy is caused by constriction of choroidal arterioles and impaired choroidal blood flow [4,6]. However, the findings in previous reports in which laser speckle flowgraphy (LSFG) was used showed that increased choroidal blood flow velocity correlated with choroidal thickening in patients with hypertensive choroidopathy, thus suggesting that choroidal hyperperfusion may be involved in hypertensive choroidopathy [7,8,35]. Those results are consistent with the increased CVI in this present study, and in particular, the increased blood flow in the choroidal outer layer may have contributed to it.

Similar to the condition reported in this study, central serous chorioretinopathy (CSC) is a disease in which SCT increases, resulting in SRD. In CSC, CVI reportedly increases during the acute phase and decreases as SRD improves [36]. In addition, in a study by Saito et al. in which LSFG was used, the authors reported increased choroidal circulation in CSC [37]. CSC is thought to be caused by increased choroidal vascular permeability and dysfunction of the RPE [38,39]. As a result, it has been reported that increased choroidal blood flow leads to thickening of the choroid, thus resulting in SRD [37]. The pathogenesis of SRD associated with preeclampsia may be similar to that of CSC, however, the difference is that CSC is associated with a topical increase in choroidal blood flow, whereas preeclampsia is associated with an increase in systemic blood flow. Thus, and as our findings illustrate, as the systemic condition calms down, the choroidal hyperperfusion improves and SRD disappears.

It has been reported that CSC is more likely to occur during the late stage of pregnancy [39], possibly due to an increase in cortisol levels in the body during the last trimester. In cases in which the patient develops SRD before delivery, it may possibly be caused by the combination of preeclampsia and an environment conducive to CSC. Reportedly, the use of cortisol medications is another risk factor for CSC [40], and the patient with SLE in this present study was taking oral prednisolone, which may have caused SRD. Since cortisol is reportedly known to reach peak levels during the last trimester of pregnancy and return rapidly to baseline after delivery [41], cortisol may not have been involved in the cases in this study that developed SRD post-birth.

It should be noted that there were several limitations to this study. First, this study was conducted at a single institution, was retrospective, and the number of cases was limited, primarily due to the difficulty of evaluating the choroid in cases prior to the development of SS-OCT. Second, since the patients in this study were either pregnant or had just given birth, and their general condition was not stable, we were not able to perform fluorescence angiography or determine hemodynamics using LSFG.

In conclusion, the findings in this study showed that SRD with preeclampsia is associated with increased thickening of the choroidal outer layer, especially in the choroidal luminal area, thus suggesting that choroidal hyperperfusion is involved in the pathogenesis of SRD with preeclampsia and that the choroid strongly reflects the systemic condition.

## Figures and Tables

**Table 1:** Patient characteristics.

No.	Age	Eye	Onset	Weeks of Pregnancy at Onset	SBP/DBP (mmHg)		BCVA (OD/OS)		Spherical Equivalent (Diopter) (OD/OS)	Complications
	(Years)				Initial Visit	Last Visit	Initial Visit	Last Visit		
1	24	B	Pre	28w0d	145/94	122/82	−0.18/0.00	−0.08/−0.08	−2.25/−2.25	SLE, APS
2	33	B	Pre	36w6d	133/72	119/72	−0.30/−0.18	−0.18/−0.18	−0.125/−0.25	
3	26	B	Post	38w6d	139/80	128/71	−0.30/0.05	−0.18/−0.08	−0.5/−1.25	
4	29	B	Post	37w2d	159/103	128/89	0.30/−0.08	−0.18/−0.18	−3.125/−3.125	Multiple Pregnancy
5	31	B	Post	33w4d	156/106	124/83	−0.08/0.05	−0.08/−0.08	−6.5/−3.625	HELLP syndrome
6	35	U	Post	27w5d	133/72	121/69	0.00/0.00	−0.08/−0.08	−0.875/−0.625	
7	37	B	Post	35w4d	145/102	122/78	0.00/−0.08	−0.08/−0.08	−4.75/−3.75	HELLP syndrome

B: bilateral; U: unilateral; Pre: pre caesarean section; Post: post caesarean section; w: weeks; d: days; SBP: systolic blood pressure; DBP: diastolic blood pressure; BCVA: the best-corrected visual acuity; OD: oculus dexter; OS: oculus sinister; SLE: systemic lupus erythematosus; APS: antiphospholipid antibody syndrome; HELLP: Hemolysis, Elevated Liver enzymes and Low Platelets.

**Table 2:** Fundus findings and changes of choroidal thickness.

No.	Eye	Serous Retinal Detachment	Elschnig’s Spots	Siegrist Streaks	Intraretinal Hemorrhages	Intraretinal Edema	Septum	Whole Layer Thickness (μm)		Inner Layer Thickness (μm)		Outer Layer Thickness (μm)		CVI (%)	
								Initial Visit	Last Visit	Initial Visit	Last Visit	Initial Visit	Last Visit	Initial Visit	Last Visit
1	OD	+	−	−	+	−	+	411	376	140	163	271	213	N/A	N/A
	OS	+	+	−	+	−	+	461	391	210	193	251	198	N/A	N/A
2	OD	+	+	+	+	−	−	518	246	312	108	206	138	68.5	66.4
	OS	+	+	−	+	−	−	536	299	234	138	302	161	65.9	63.9
3	OD	+	−	−	−	−	+	381	286	80	80	301	206	68.7	66
	OS	+	−	−	−	−	+	420	302	143	102	277	200	65.7	64.4
4	OD	+	−	−	+	+	−	N/A	N/A	N/A	N/A	N/A	N/A	N/A	N/A
	OS	+	−	−	+	+	−	N/A	N/A	N/A	N/A	N/A	N/A	N/A	N/A
5	OD	+	+	+	−	−	−	400	256	131	138	269	118	N/A	N/A
	OS	+	+	+	−	+	+	455	240	130	115	325	125	N/A	N/A
6	OD	-	-	-	-	-	-	N/A	N/A	N/A	N/A	N/A	N/A	N/A	N/A
	OS	+	+	−	+	−	−	N/A	N/A	N/A	N/A	N/A	N/A	N/A	N/A
7	OD	+	−	−	−	−	−	354	240	103	91	251	149	67.9	65.2
	OS	+	−	−	−	+	−	308	224	144	95	164	129	66.7	66.4
Average								424.4	286	162.7	122.3	261.7	163.7	67.2	65.4

CVI: choroidal vascularity index; N/A means not available.

## Data Availability

The data in this study is available upon a reasonable request to the corresponding author, as it is not publicly available due to privacy reasons and ethical concerns.
